# Impact of bone cement leakage on early postoperative pain in percutaneous vertebroplasty for osteoporotic vertebral compression fractures

**DOI:** 10.3389/fsurg.2026.1830335

**Published:** 2026-05-18

**Authors:** Can Cao, Zenghui Zhao, Xianda Gao, Di Zhang

**Affiliations:** 1 Department of Spinal Surgery, The Third Hospital of Hebei Medical University, Shijiazhuang, Hebei, China; 2 Department of Orthopedics, Hebei General Hospital, Shijiazhuang, Hebei, China

**Keywords:** bone cement leakage, osteoporotic vertebral compression fracture, percutaneous vertebroplasty, postoperative pain, risk factors

## Abstract

**Objective:**

This study aimed to explore the impact of bone cement leakage and its distribution on early postoperative pain following percutaneous vertebroplasty (PVP) for osteoporotic vertebral compression fractures (OVCFs) and to determine whether bone cement leakage is an independent risk factor for early postoperative pain.

**Methods:**

A retrospective analysis was conducted on 262 patients with OVCF who underwent PVP at the Third Hospital of Hebei Medical University between December 2020 and December 2023. Preoperative and postoperative imaging was used to assess bone cement leakage. Clinical data were collected, and pain intensity was evaluated using Visual Analog Scale (VAS) scores at preoperative, postoperative, and last follow-up visits. Univariate analysis was used to screen potential risk factors, and multivariate logistic regression analysis was performed to identify independent risk factors for early postoperative pain.

**Results:**

Bone cement leakage occurred in 68 patients (25.95%). Postoperative VAS scores were significantly higher in the bone cement leakage group (BCL) than in the non-leakage group (NBCL) (3.99 ± 1.77 vs. 2.20 ± 1.30, *p* < 0.001). Multivariate logistic regression analysis identified bone cement distribution (OR = 11.525, 95% CI = 3.730–35.610, *p* < 0.001) and bone cement leakage (OR = 10.167, 95% CI = 5.062–20.420, *p* < 0.001) as independent risk factors for early postoperative pain.

**Conclusion:**

This study demonstrates that bone cement leakage and its distribution are closely associated with early postoperative pain following PVP. Bone cement leakage is an important risk factor and should be closely monitored for early identification and management.

## 1 Introduction

Percutaneous vertebroplasty (PVP) is a widely used minimally invasive treatment for patients with osteoporotic vertebral compression fractures (OVCFs) (1). Due to its low invasiveness and quick recovery, PVP has become a common choice for managing vertebral compression fractures in elderly patients with osteoporosis (2). This procedure stabilizes the affected vertebrae, alleviates pain, and improves the patient's quality of life by injecting bone cement (3). However, despite the significant effectiveness of PVP in symptom relief, potential complications may still arise during the use of bone cement, particularly the occurrence of cement leakage (4).

Bone cement leakage refers to the accidental overflow of bone cement beyond the vertebral boundaries into the surrounding tissues during the injection process. This issue is not uncommon in PVP treatment, with reported incidence rates varying across different studies, reaching up to 75% (5). Cement leakage can lead to severe complications, such as spinal cord compression, nerve root injury, pulmonary embolism, and may also be closely related to increased postoperative pain (6–8). However, the mechanisms behind cement leakage and its specific impact on postoperative outcomes remain insufficiently studied (9).

The occurrence of cement leakage is closely related to multiple factors, including injection pressure, vertebral bone quality, and technical aspects of the surgical procedure. Clinically, some patients may not experience significant symptoms postoperatively, but existing studies have suggested a correlation between cement leakage and adverse outcomes, such as postoperative pain and neurological dysfunction. The onset of pain after PVP is a complex, multifactorial process that may involve multiple pathophysiological mechanisms. Common sources of pain include the local effects of cement injection on vertebral structures, the distribution of cement within the vertebra, and nerve damage or local inflammatory responses caused by cement leakage.

Previous research has indicated that uneven cement distribution within the vertebra, particularly leakage into the spinal canal or neural foramina, may be closely associated with persistent or intensified postoperative pain. Furthermore, the frequency and extent of cement leakage are considered important predictive factors for postoperative pain. However, comprehensive research on the effects of cement leakage and its distribution on early postoperative pain following PVP is still lacking.

This study aimed to explore, through retrospective analysis, whether cement leakage and its distribution within the vertebra are risk factors for early postoperative pain following PVP. By collecting clinical case data, this research sought to analyze the relationship between cement leakage and postoperative pain, providing strong evidence for the early identification and management of patients at risk.

## 2 Materials and methods

### 2.1 Patients

A total of 262 patients who underwent PVP between December 2020 and December 2023 at the Third Hospital of Hebei Medical University in China were reviewed retrospectively. The inclusion criteria were as follows: (1) diagnosed osteoporosis (*T* ≤ −2.5 by DEXA); (2) imaging-confirmed single-segment OVCF in the thoracic or lumbar spine (defined as a compression fracture involving only one vertebral body confirmed by imaging, without additional vertebral fractures); (3) acute or subacute fracture confirmed by magnetic resonance imaging (MRI); (4) underwent unilateral PVP; and (5) minimum follow-up of 12 months. The exclusion criteria were as follows: (1) spinal tumors, infections, or other non-osteoporotic diseases; (2) presence of neurological compression symptoms; (3) coexisting fragility fractures; (4) new vertebral fractures during follow-up; (5) incomplete clinical data; and (6) high-energy violent fracture.

### 2.2 Data analysis

Preoperative evaluation included anterior–posterior and lateral x-rays, computed tomography, and 1.5-T MRI. Postoperative anterior–posterior and lateral x-rays were used to determine bone cement leakage, which was classified into four types: (1) intervertebral space leakage; (2) spinal canal leakage; (3) perivertebral space leakage, including anterior and lateral leakage; and (4) mixed type.

Patient characteristic including age, gender, body mass index (BMI), bone mineral density, smoking status, drinking status, history of symptoms, and standardized treatment for osteoporosis during follow-up period were recorded. BMD was detected by dual energy x-ray absorptiometry (DEXA) with *T* ≤ −2.5 confirming osteoporosis. History of symptoms was calculated from onset of the symptoms to surgery. Imaging measurement data including local kyphosis Cobb angle, lumbar lordosis (LL), and thoracic kyphosis (TK) were measured three times, and the average values were recorded for further analysis. Local kyphosis Cobb angle was defined as the angle between the upper endplate of the cranial vertebral body and the lower endplate of the caudal vertebral body of the compressed vertebra. LL was defined as the angle between the upper endplate of L1 and the lower endplate of L5. TK was defined as angle between the upper endplate of T4 and the lower endplate of T12. Surgical data included surgical segment, surgical time, volume of bone cement, bone cement distribution, and leakage. Intravascular leakage was also reviewed on postoperative imaging; however, no such cases were identified in this study. Bone cement distribution included two types: (1) Type 1, bone cement failed to touch the midline of the vertebral body according to anterior–posterior x-ray; (2) Type 2, bone cement crossed the midline of the vertebral body according to anterior–posterior x-rays. Visual Analog Scale (VAS) scores (0–10) were used to assess spinal pain at preoperative, postoperative, and last follow-up visit. Postoperative VAS scores were obtained 1 month after surgery. Early postoperative pain was defined as VAS >3 at 1 month after surgery. This threshold was chosen based on previous studies suggesting that a VAS score >3 represents clinically meaningful pain requiring intervention (10). Standardized treatment for osteoporosis included anti-osteoporosis medications such as bisphosphonates, calcium supplementation, and vitamin D.

### 2.3 Statistical analysis

The SPSS program (version 27.0; SPSS Inc., Chicago, IL, USA) was used for statistical analyses. A *p-*value < 0.05 was considered statistically significant. Quantitative data between two groups were compared using Student's *t*-test or the Mann–Whitney *U*-test. Preoperative, postoperative, and last follow-up VAS scores in each group were tested using the Friedman test according to non-normality. Next, we will conduct multiple comparisons based on the above VAS score results. These comparisons will be performed when *p* < 0.05 in the Friedman test. The Friedman test will be followed by Wilcoxon tests, and the p - value will be corrected to 0.0167 according to the Bonferroni correction to decrease the type 1 error. Potential risk factors for early back pain after surgery were initially screened using univariate analysis. The factors with *p* < 0.10 in univariate analysis were further tested using multivariate logistic regression analysis with adjusted odds ratios (ORs), 95% confidence intervals (CIs), and corresponding *p* values.

## 3 Results

### 3.1 Patient characteristics and grouping

**Figure 1:**
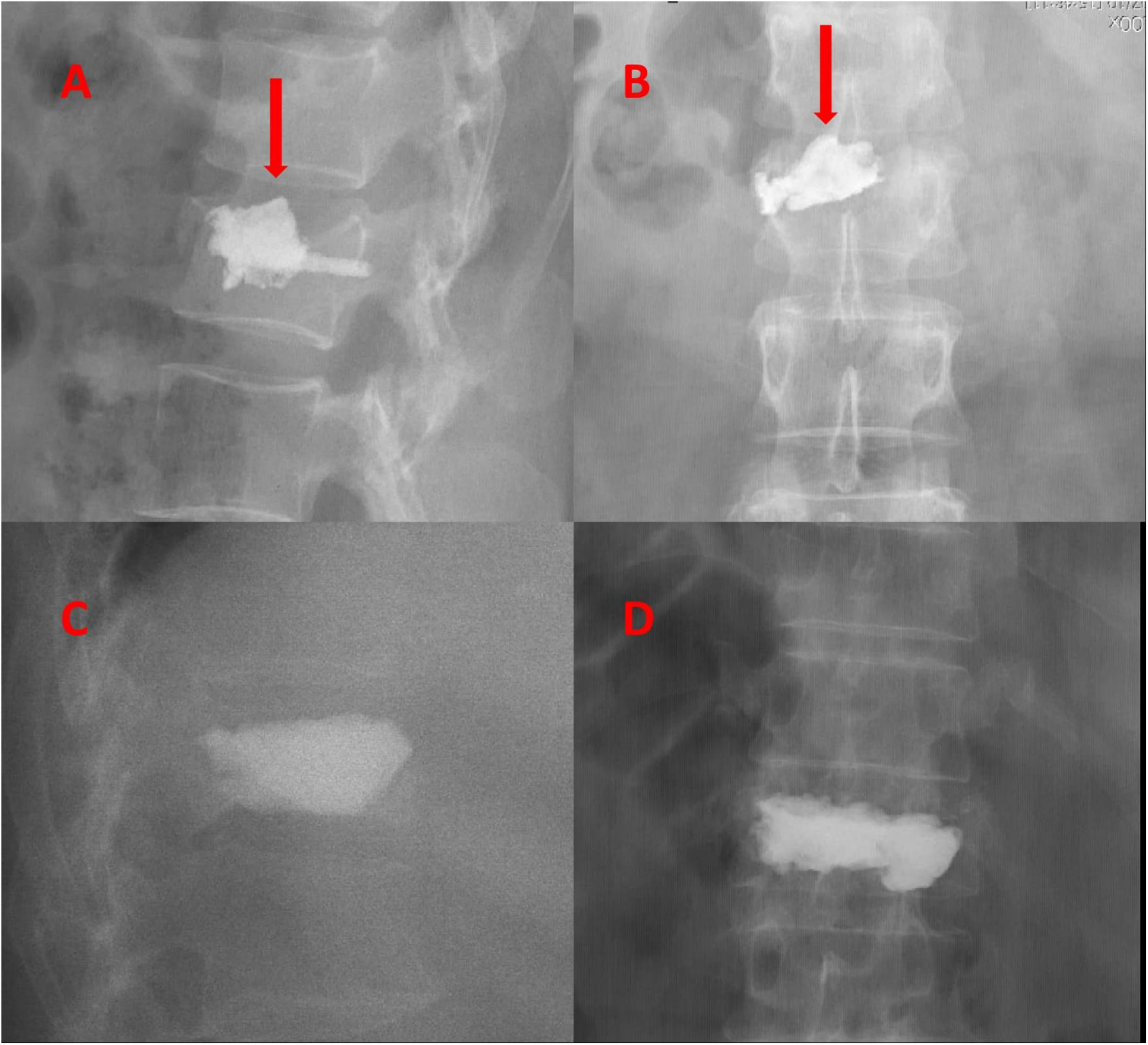
**(A,B)** Postoperative anteroposterior and lateral x-ray of the vertebral body showing cement leakage into the intervertebral space, with the patient reporting early postoperative low back pain. **(C,D)** Postoperative anteroposterior and lateral x-ray of the vertebral body showing good distribution and positioning of the bone cement, with the patient reporting the disappearance of pain symptoms.

A total of 262 patients were reviewed retrospectively in the study. Based on postoperative x-rays, bone cement leakage was found in 68 patients (25.95%), who were classified into the bone cement leakage group (Group BCL), while the remaining 194 patients were divided into the no bone cement leakage group (Group NBCL) (Figure 1). There were no statistical differences found in age (*p* = 0.781), gender (*p* = 0.223), BMI (*p* = 0.522), smoking status (*p* = 0.608), and drinking status (*p* = 0.247) between the groups. The BMD was −3.27 ± 0.47 in Group BCL and −3.23 ± 0.56 in Group NBCL, showing no statistical differences (*p* = 0.359). History of symptoms (*p* = 0.537) and follow-up period (*p* = 0.743) also showed no statistical differences between the groups. After surgery, 45 patients (66.18%) in Group BCL and 134 patients (69.07%) in Group NBCL received standardized treatment for osteoporosis, with no statistical differences between groups (*p* = 0.659).

### 3.2 Imaging measurement data and surgical date between two groups

In Group BCL, local kyphosis Cobb angle, LL, and TK were 10.40 ± 6.67, 24.68 ± 10.54, and 42.71 ± 12.76, respectively, and in Group NBCL, these values were 9.89 ± 6.93, 24.81 ± 9.42, and 41.06 ± 12.33, showing no statistical differences (local kyphosis Cobb angle, *p* = 0.449; LL, *p* = 0.789; TK, *p* = 0.290). There were also no statistical differences (*p* = 0.701) found in surgical segment between the groups. The surgical time in Group BCL was 34.63 ± 11.21 min, which was longer (*p* = 0.039) than 31.47 ± 11.74 min in Group NBCL. The volume of bone cement showed no statistical differences (*p* = 0.110); however the standard deviation in Group BCL (1.83) was clearly larger than that in Group BCL (1.18), which indicated that data dispersion in Group BCL was greater. According to classification of bone cement distribution, there were 10 type 1 (16.18%) and 58 type 2 in Group BCL and 11 type 1 (5.67%) and 183 type 2 in Group NBCL, which showed significant statistical differences (*p* = 0.018). In Group BCL, postoperative x-rays showed intervertebral space leakage in 42 patients (61.76%), spinal canal leakage in 16 patients (23.53%), perivertebral space leakage in 7 patients (10.29%), and mixed type in 3 patients (4.41%).

### 3.3 Clinical outcomes and risk factors for early back pain

No serious complications were found in the 262 patients following PVP. In Group NBCL, the preoperative VAS score was 7.71 ± 1.40, which significantly decreased to 2.20 ± 1.30 postoperatively (*p* < 0.001) and 1.71 ± 1.08 at last follow-up (*p* < 0.001). Similarly, in Group BCL, the preoperative VAS score significantly decreased to 3.99 ± 1.77 postoperatively (*p* < 0.001) and 1.87 ± 1.01 at last follow-up (*p* < 0.001). Between groups, no statistical differences were observed in preoperative (*p* = 0.251) or last follow-up (*p* = 0.206) VAS scores. However, postoperative VAS scores in Group BCL were significantly higher than those in Group NBCL (*p* < 0.001), which indicated that patients with bone cement leakage experienced more early back pain after PVP.

According to postoperative VAS scores, early back pain was defined as a score greater than 3. Logistic regression analysis was performed to identify the risk factors for early back pain after PVP. Univariate analysis showed that bone cement distribution (*p* < 0.001) and bone cement leakage (*p* < 0.001) were associated with early back pain. Multivariate logistic regression confirmed that bone cement distribution (OR = 11.525, 95% CI = 3.730–35.610, *p* < 0.001) and bone cement leakage (OR = 10.167, 95% CI = 5.062–20.420, *p* < 0.001) were risk factors.

## 4 Discussion

Percutaneous vertebroplasty is a minimally invasive surgery widely used for treating osteoporotic vertebral compression fractures due to its small incision size and rapid recovery (9). However, cement leakage is a common complication, with studies reporting varying incidences, some as high as 75% (5). In this study, although there were no significant differences in the overall patient characteristics between the cement leakage and non-leakage groups, the use and distribution of bone cement during surgery still showed a certain risk correlation. This suggests that cement leakage, as a common surgical complication, should not be overlooked given its potential impact on outcomes (8). Ren et al. noted that “the volume of injected bone cement” and injection handling technique are independent risk factors for leakage (11). Although the volume of bone cement was not significantly different between the two groups in this study, previous studies have suggested that excessive cement volume may increase the risk of leakage. The lack of statistical significance in our study may be related to the relatively standardized surgical procedures and controlled injection techniques. In addition, the greater standard deviation observed in the leakage group suggests higher variability, which may indicate that extreme values rather than average volume contribute to leakage risk. Tang et al. also demonstrated that the distribution type of bone cement (solid vs. loose) has a significant impact on different types of leakage (9).

In our cement leakage classification, “leakage into the intervertebral disc” was the most common type, occurring in 42 cases, accounting for approximately 62% of the 68 cases in the cement leakage group. This phenomenon indicates that the intervertebral disc, as a relatively “weak link” in the anatomical structure, is more prone to leakage during the cement injection process. Sun et al., in a mid-term follow-up study, found that disc-type leakage was more common among various types and that patients showed no obvious symptoms of nerve root injury (12). Another imaging study indicated that the incidence of disc leakage was significantly higher in cases with vertebral fissures (13). In contrast, other leakage types—such as spinal canal, vertebral body exterior, and mixed leakage—though less frequent, may still cause varying degrees of nerve root compression and inflammatory reactions, affecting the patient's postoperative recovery. Intravertebral disc leakage of bone cement can lead to disc degeneration and may affect postoperative recovery. Mao et al., in an animal study, confirmed that PMMA infiltration into the intervertebral disc can induce significant disc degeneration, accompanied by structural tissue changes and cellular apoptosis (14).

Table 1 shows no significant statistical differences in basic clinical characteristics such as age, gender, BMI, bone mineral density, smoking status, and drinking status between the two groups, suggesting that the baseline conditions of patients were relatively balanced and postoperative differences between the groups are unlikely to be explained by these factors. As shown in Table 2, the operative time in the leakage group (34.63 ± 11.21 min) was significantly longer than in the non-leakage group (31.47 ± 11.74 min) (*p* = 0.039), suggesting that the complexity of the procedure or technical details might influence the occurrence of cement leakage, or that the surgeon's response time in decision-making and handling the situation during cement leakage may contribute to the difference. Cement distribution type also showed significant differences between the two groups (*p* = 0.018), with distribution in the leakage group more likely to be Type 1 (cement distribution not crossing the midline on the coronal view). This phenomenon is likely because surgery was terminated earlier once leakage was detected, to avoid other adverse events. Liang et al. reported that bone cement distribution ratio and vertebral wall rupture are significantly associated with the risk of leakage. A higher proportion of bone cement filling is more likely to cause leakage, while prematurely stopping the injection may result in asymmetric distribution (15).

**Table 1:** Comparison of patient characteristics between group BCL and group NBCL.

Variable	Group BCL (*n* = 68)	Group NBCL (*n* = 194)	*t*/*z*/*χ*^2^	*p*-value
Age (years)	70.60 ± 6.11	70.84 ± 6.11	0.278	0.781
Gender			1.485	0.223
Male	10	18		
Female	58	176		
Body mass index	23.55 ± 4.53	23.94 ± 4.35	0.641	0.522
Bone mineral density	−3.27 ± 0.47	−3.23 ± 0.56	0.918	0.359
Smoking			0.263	0.608
Yes	7	16		
No	61	178		
Drinking			1.340	0.247
Yes	24	54		
No	44	140		
History of symptoms (days)	14.09 ± 10.88	14.53 ± 13.66	0.617	0.537
Follow-up period (months)	17.38 ± 7.94	17.51 ± 7.75	0.328	0.743
Local kyphosis Cobb angle	10.40 ± 6.67	9.89 ± 6.93	0.757	0.449
Lumbar lordosis	24.68 ± 10.54	24.81 ± 9.42	0.256	0.798
Thoracic kyphosis	42.71 ± 12.76	41.06 ± 12.33	1.059	0.290
Standardized treatment for osteoporosis			0.195	0.659
Yes	45	134		
No	23	60		

**Table 2:** Comparison of surgical data between group BCL and group NBCL.

Variable	Group BCL (*n* = 68)	Group NBCL (*n* = 194)	*t*/*z*/*χ*^2^	*p*-value
Surgical segment			0.710	0.701
T7–10	13	43		
T11–L2	36	106		
L3–5	19	45		
Surgical time (min)	34.63 ± 11.21	31.47 ± 11.74	2.068	0.039
Volume of bone cement (mL)	5.47 ± 1.83	5.23 ± 1.18	1.599	0.110
Distribution of cement bone			5.576	0.018
Type 1	10	11		
Type 2	58	183		
Subclassification of cement bone leakage				
Intervertebral space	42	–		
Cerebrospinal canal	16	–		
Extravertebral	7	–		
Mixed type	3	–		

Table 3 shows that although there were no significant differences in preoperative VAS scores between the two groups, the postoperative VAS score in the leakage group was significantly higher than in the non-leakage group (3.99 ± 1.77 vs. 2.20 ± 1.30, *p* < 0.001). This is consistent with the findings of Li et al., who observed that patients in the leakage group generally had higher VAS scores after vertebroplasty, supporting the negative impact of bone cement leakage on postoperative pain (16). However, this difference disappeared at the final follow-up. This result indicates that cement leakage has a significant adverse impact on early postoperative pain, possibly related to local tissue irritation, nerve root compression, or local inflammatory reactions. However, as time progressed, pain improved in both groups, suggesting that while leakage may exacerbate early postoperative pain, its long-term effects may diminish through the body's self-regulation and recovery (13).

**Table 3:** Clinical efficacy of bone cement leakage.

Variable	Group BCL (*n* = 68)	Group NBCL (*n* = 194)	*z*	*p*-value
VAS score				
Preoperative	7.91 ± 1.37	7.71 ± 1.40	1.149	0.251
Postoperative	3.99 ± 1.77*	2.20 ± 1.30*	7.912	<0.001*
Last follow-up	1.87 ± 1.01*	1.71 ± 1.08*	1.266	0.206

* Significantly different from preoperative (*p* < 0.0167).

Table 4 confirms through multivariable logistic regression that cement leakage (adjusted OR = 10.167, 95% CI: 5.062–20.420, *p* = 0.000) and cement distribution (adjusted OR = 11.525, 95% CI: 3.730–35.610, *p* = 0.000) are independent risk factors for early postoperative pain following PVP.

**Table 4:** Factors related to early pain after PVP: multiple logistic regression analysis.

Variable	Adjusted odds radio	95% Confidence interval	*p*-value
Distribution of cement bone	11.525	3.730–35.610	0.000
Leakage of cement bone	10.167	5.062–20.420	0.000

From a clinical perspective, several measures may help reduce the risk of cement leakage during PVP: careful preoperative evaluation of fracture morphology and posterior vertebral wall integrity; selection of an appropriate puncture trajectory; injection during the doughy phase of cement viscosity; slow and incremental injection under continuous fluoroscopic monitoring; immediate cessation of injection once leakage is suspected; and avoidance of excessive cement filling solely to achieve wider distribution.

This study has several limitations. As this study is a retrospective study, the data collection and processing may involve specific selection biases. Although the overall sample size was acceptable, the number of cases in each classification type was relatively small, potentially affecting the statistical power of the classification analysis. Because low back pain is a nonspecific symptom, unmeasured musculoskeletal or degenerative conditions may still have influenced postoperative pain assessment. Furthermore, the follow-up period was relatively short, making it difficult to fully assess long-term clinical efficacy and complications. Different surgical techniques, injection devices, and operator experience may influence cement distribution and leakage rates, but this study did not fully control for these variables, which should be further considered in future research. Different surgical techniques may exhibit variations in bone cement distribution and leakage risk. PKP may reduce certain leakage risks due to the formation of cavities during balloon dilation, but further prospective comparative studies are still required for confirmation.

## 5 Conclusion

Percutaneous vertebroplasty is an effective treatment for osteoporotic vertebral compression fractures. However, bone cement leakage—a common complication—has a significant impact on early postoperative pain. This retrospective analysis found a clear association between bone cement leakage and early postoperative pain. Multivariate regression analysis confirmed that bone cement leakage was a risk factor for early postoperative pain. In addition, the distribution pattern of bone cement was also found to be related to early postoperative pain.

## Data Availability

The original contributions presented in the study are included in the article/Supplementary Material; further inquiries can be directed to the corresponding authors.
